# Vulvo-Vaginal-Oral Lichen Planus: A Case Report and Literature Review

**DOI:** 10.7759/cureus.75204

**Published:** 2024-12-06

**Authors:** Khalid F Almutairi, Sarah B Alshammari, Faisal Almutairi, Abdulrahman Almujalli, Roba Alsahman, Asmaa Faden

**Affiliations:** 1 College of Dentistry, Majmaah University, Al Majma'ah, SAU; 2 College of Dentistry, King Saud University, Riyadh, SAU; 3 Department of General Dentistry, King Saud University, Riyadh, SAU; 4 Department of Dentistry, University of Dundee, Dundee, GBR; 5 Department of Dentistry, Sheffield University Dental School, Sheffield, GBR; 6 Oral Medicine and Diagnostic Science, College of Dentistry, King Saud University, Riyadh, SAU

**Keywords:** genital mucosa, lichen planus, oral lichen planus, rare disease, ulcerative lesions

## Abstract

Vulvo-vaginal-oral lichen planus (VVO-LP) is a chronic inflammatory condition affecting the mucous membranes of the oral cavity, skin, and genital areas. The exact etiology remains unclear, although immune-mediated mechanisms are considered likely contributors. It is a rare form of lichen planus, which typically presents in adults and is more common in middle-aged women. Key clinical features include painful ulcerative lesions in the oral, vaginal, and cutaneous regions, often resistant to conventional treatment. This condition can significantly impact the patient's quality of life due to persistent symptoms and complications such as scarring and infection.

In this review, we present a case of a 43-year-old Saudi female who presented to our Oral Medicine Clinic with recurrent painful ulcerative lesions affecting the mouth, skin, and vaginal mucosa. Her medical history included type II diabetes mellitus and hypercholesterolemia. Oral biopsy results confirmed the diagnosis of lichen planus, with no signs of dysplasia. The patient was treated with a variety of topical and systemic therapies, including corticosteroids and immunosuppressants, but showed only temporary improvement. Despite short-term remission with all provided medications, the lesions recurred after discontinuation of treatment in a more severe form. This case report emphasizes the challenges associated with the diagnosis and management of VVO-LP, particularly its resistance to standard therapies. The patient's case underscores the need for further research into novel treatment strategies and long-term management plans for this rare and debilitating condition.

## Introduction

Lichen planus (LP) is a T-cell-mediated inflammatory dermatosis that affects both keratinized and non-keratinized squamous epithelium [1]. It is a chronic inflammatory disease that affects the nails, scalp, skin (cutaneous LP), and mucous membranes (oral, genital, or esophageal). Oral LP (OLP) is more common in women than in men, with a prevalence of 1% to 2% in individuals over 15 years of age, and is frequently observed in the general population, particularly among people in their 5th and 6th decades of life [2,3]. Additionally, the genital mucous membranes may also be affected alongside the oral mucosa and skin [4]. It has been reported that genital LP develops in about 20% of women with OLP [5].

Although the exact etiology remains undetermined, an autoimmune mechanism has been hypothesized. Several reports in recent years have shown a correlation between LP and liver damage caused by hepatitis C [3]. Though its exact cause is unknown, many studies support the hypothesis that it is a complex immunologic disease mediated by cytotoxic T cells directed against basilar keratinocytes [6].

Lucchese A et al. discovered a potential link, noting that scarring occurs in 90% of patients with vulvovaginal-gingival lichen planus (VVG-LP), a phenomenon that is rare in other forms of LP [5]. VVG-LP presents with typical oral manifestations of LP in the oral cavity [7]. In the genital area, LP can manifest in three reported forms: erosive, classic, and hypertrophic [8,9].

Genital erosive lichen planus (GELP) is characterized by painful and scarring vulvo-vaginal erosions, and in some cases, this leads to the absorption of the labia and stenosis or total obliteration of the vagina [10]. A more recent publication, however, found histologically confirmed vulval LP in 57% of 42 female patients with OLP [11].

As for the most appropriate tests to obtain a definitive diagnosis, they include a biopsy for routine H&E staining and direct immunofluorescence (DIF). A histopathological analysis of a biopsy taken from the peri-lesional tissue and another peri-lesional biopsy to rule out other vesiculobullous diseases is also recommended [3,12,13]. The diagnosis is further supported by clinical presentation and/or characteristic histologic findings, such as the presence of a well-defined inflammatory band in the superficial connective tissue at the dermoepidermal junction, an inflammatory band predominantly consisting of lymphocytes, signs of basal cell layer degeneration, e.g., Civatte bodies, abnormal keratinocytes, or basal apoptosis [14].

Regarding the management of LP, after establishing the definitive diagnosis, no cure has been found for LP, similar to many autoimmune diseases. However, the treatment goal is to control symptoms such as pain, erythema, or ulceration and to monitor the lesions for any dysplastic changes. Thus, multiple management methods have been used with various reported responses. The first-line treatment is corticosteroids with applications including topical, systemic, or intralesional injections [15]. Topical application of high-potency corticosteroids, such as clobetasol propionate 0.05%, is usually necessary throughout life. Some patients benefit from the addition of topical tacrolimus [16]. Second-line treatments for OLP include systemic immunosuppressants or immune-modifying agents such as retinoids, methotrexate, cyclophosphamide, azathioprine, calcineurin inhibitors, and mycophenolate mofetil [17,18]. Neglecting the symptoms or not administering appropriate treatment can exacerbate the patient's condition. Recent systematic reviews indicate that none of the available therapeutic interventions effectively manage symptomatic OLP, and there is no definitive evidence supporting the superiority of any specific treatment approach [19].

Lucchese A et al. in 2016 noted that OLP lesions are typically symmetrical and bilateral, with the buccal mucosa commonly involved. In the literature, the condition was referred to as VVGLP when it primarily affected the gingiva. However, in our report, the tongue, buccal mucosa, and gingiva were all affected, leading to the use of the term VVOLP, which refers to VVO-LP [20].

## Case presentation

A 43-year-old Saudi female, married with two children, presented on March 3, 2021, to our Oral Medicine Clinic with complaints of persistent ulcerative lesions affecting the oral cavity, skin, and genital area. According to the referral report, she had white patches on both sides of the buccal mucosa. Despite previous treatments with corticosteroid creams prescribed by her dentist, her symptoms persisted. The patient’s medical history included type II diabetes mellitus and hypercholesterolemia, managed with Glucophage and Lipitor. She denied any history of smoking or alcohol use.

**Figure 1:**
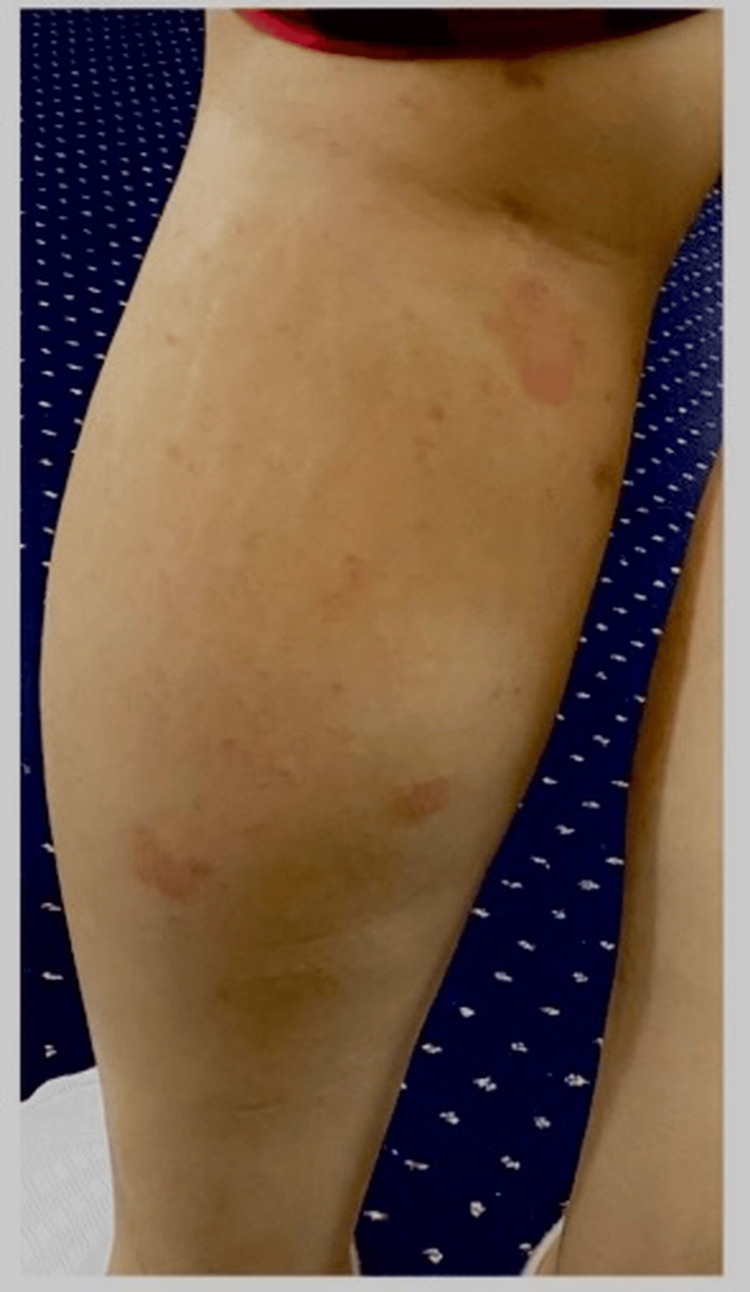
Small ulcerations with crusting observed on the cuffs of the legs.

**Figure 2:**
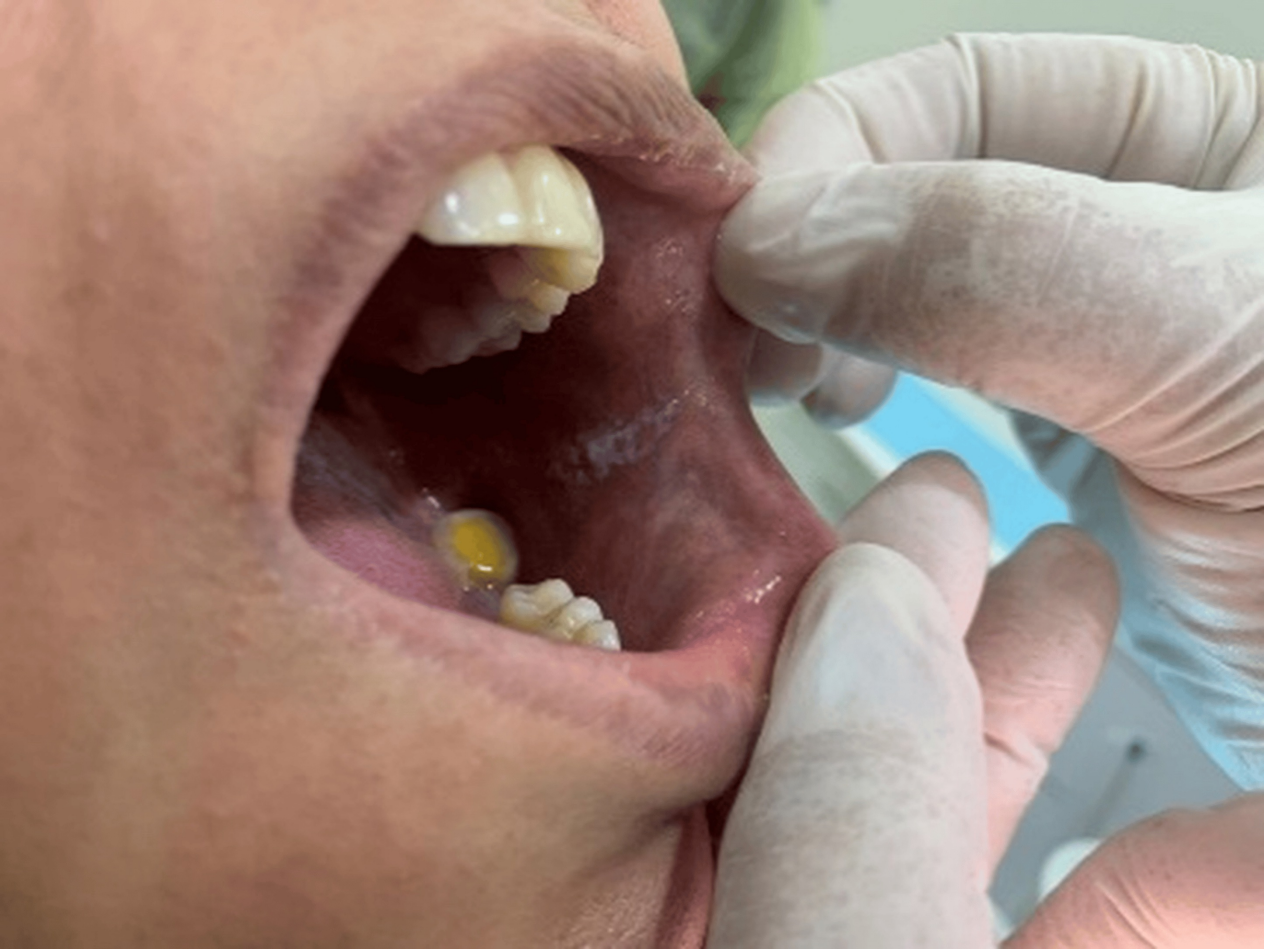
Bilateral mixed red and white striated lesions with black pigmentation were observed on the buccal mucosa.

**Figure 3:**
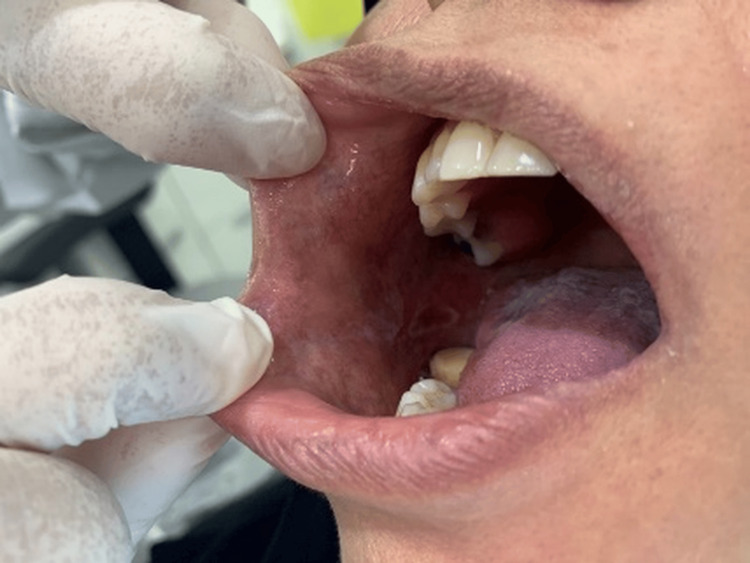
Bilateral mixed red and white striated lesions with black pigmentation were observed on the buccal mucosa.

**Figure 4:**
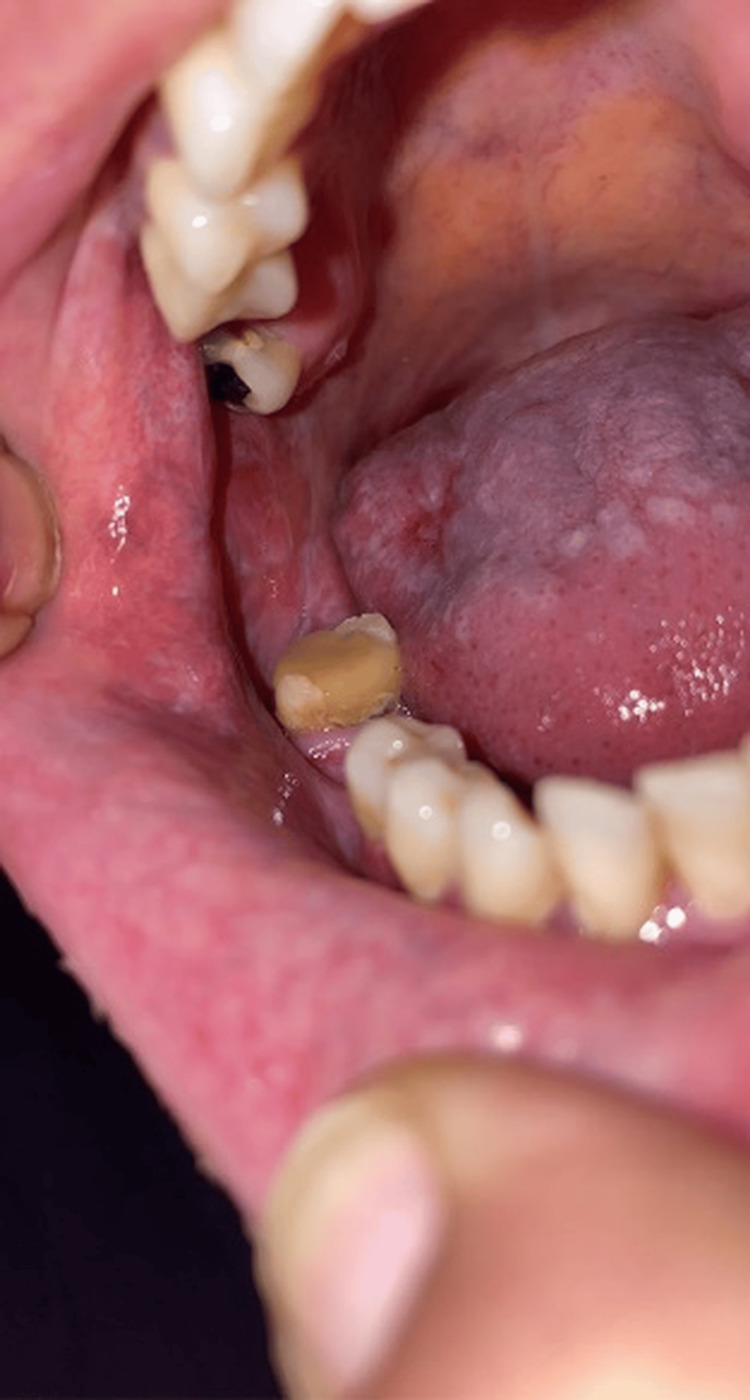
Bilateral mixed red and white striated lesions with black pigmentation observed on the buccal mucosa.

**Figure 5:**
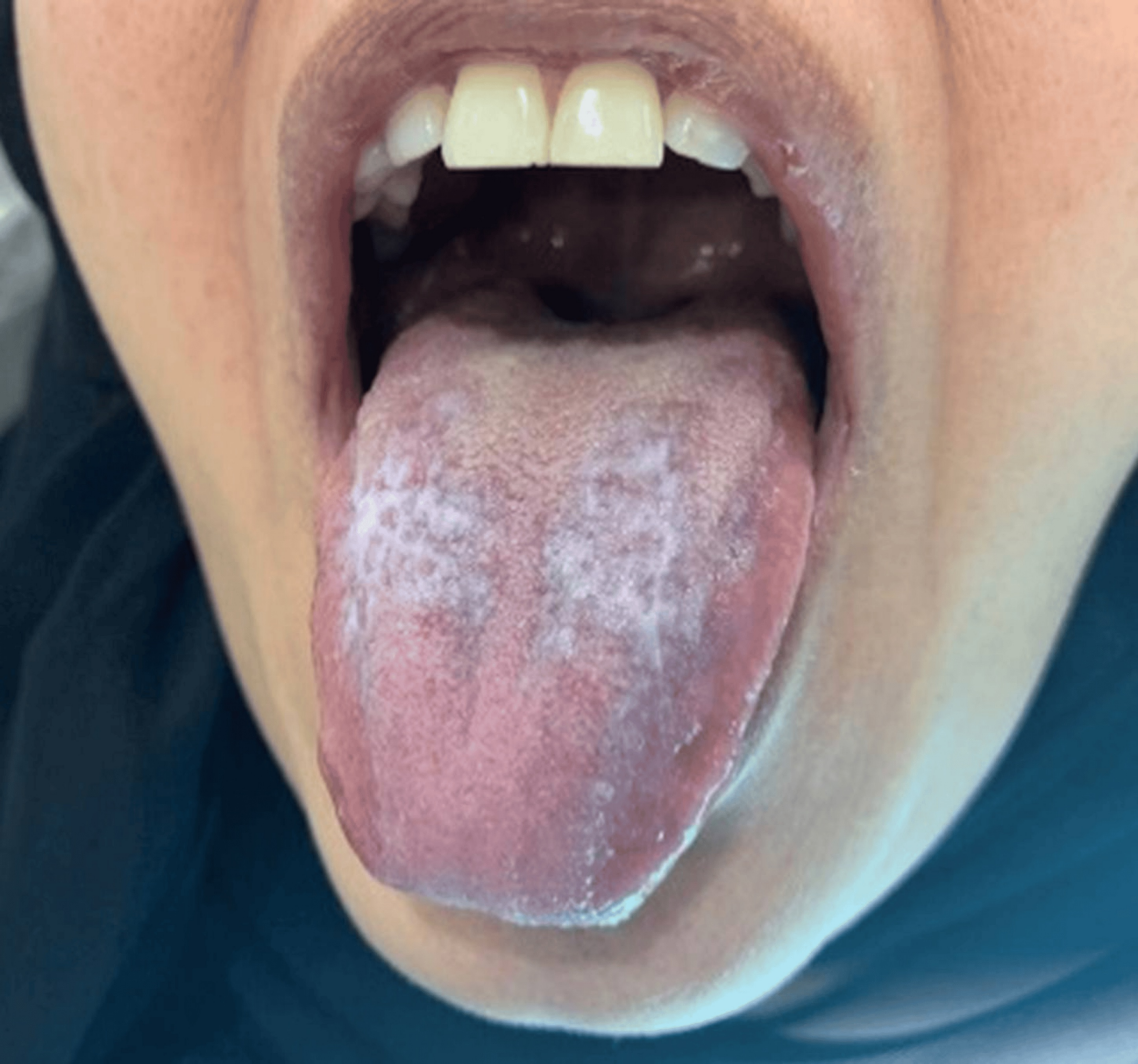
Post-inflammatory hyperpigmentation was observed on the dorsum of the tongue.

Extraoral clinical examination revealed small ulcerations with crusting on the thighs and cuffs of the legs (Figure 1), which had recently spread to the genital area. No significant lymphadenopathy was detected. Intraoral examination revealed bilateral mixed red and white lesions on the buccal mucosa with striated black pigmentation defined as post-inflammatory hyperpigmentations (Figures 2-4) and post-inflammatory pigmentation was also noted on the dorsum of the tongue (Figure 5).

**Figure 6:**
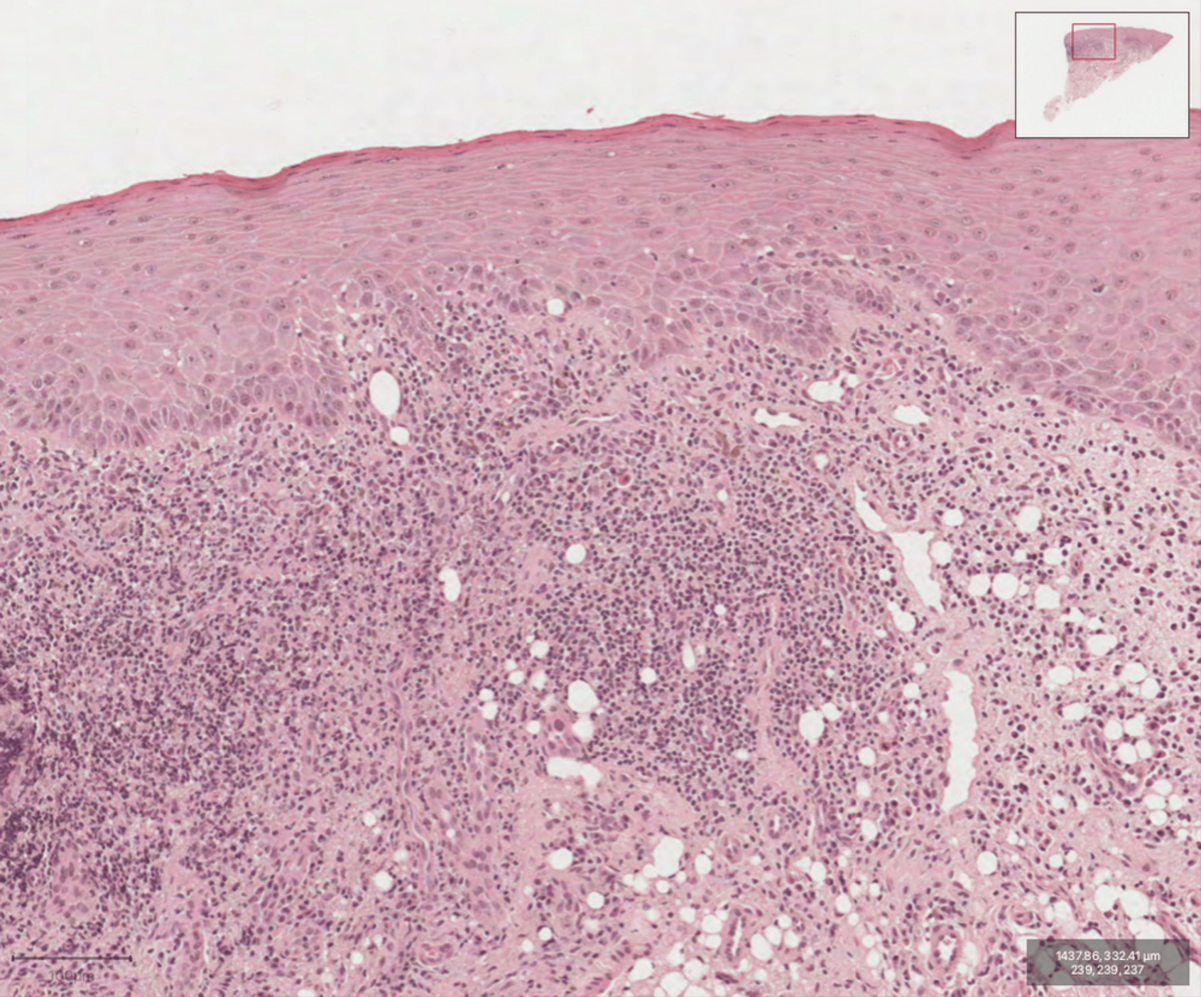
The biopsy of the mucosal lesion revealed keratosis, mild spongiosis, and a lichenoid band-like chronic inflammation with scattered foci of Civatte body formation within the squamous epithelium.

An intraoral biopsy confirmed lichen planus, with findings of keratosis, mild spongiosis, and a lichenoid band-like inflammation with Civatte bodies. No dysplasia was observed (Figure 6).

Laboratory tests were ordered to rule out systemic conditions that could exacerbate her symptoms (Table 1).

**Table 1:** Laboratory investigations. IgD: immunoglobulin D; mg/dL: milligrams per deciliter; IgA: immunoglobulin A; IgG: Immunoglobulin G; IgE: Immunoglobulin E; IU/mL: International units per milliliter; IgM: immunoglobulin M; g/L: Grams per liter; Baso: Basophils; Mono: Monocytes; Neut: Neutrophils; x10^9/L: Cells per liter in billions; Lymph: Lymphocytes; Eos: Eosinophils; Hgb: Hemoglobin; x10^12/L: Cells per liter in trillions; Hct: Hematocrit; L/L: Liters per liter; MCV: Mean corpuscular volume; fL: Femtoliters; MCH: Mean corpuscular hemoglobin; pg: Picograms; MCHC: Mean corpuscular hemoglobin concentration; RDW: Red cell distribution width; NRBC: Nucleated red blood cells; MPV: Mean platelet volume; Alk Phos: Alkaline phosphatase; U/L: Units per liter; Total Protein: Total protein; mmol/L: Millimoles per liter; Glu F: Fasting glucose; µmol/L: Micromoles per liter; CO2: Bicarbonate; BUN: Blood urea nitrogen; ALT: Alanine aminotransferase; AST: Aspartate aminotransferase; AGAP: Anion gap.

Laboratory test	Value (reference range)
Immunoglobulin D, Quantitative, Serum	Less than 4 mg/dL (1-10)
Immunoglobulin A, IgA	202 mg/dL (70-400)
Immunoglobulin G, IgG	1105.380 mg/dL (700-1600)
Immunoglobulin E, Serum	14.4 IU/mL (0-100)
Immunoglobulin M, IgM Serum	0.55 g/L (0.4-2.3)
Baso (Basophils)	0.00 % (0-0.1)
Mono (Monocytes)	0.34 % (0.1-1.1)
Neut (Neutrophils)	1.66 x10^9/L (2-7.5) Low
Lymph (Lymphocytes)	1.95x10^9/L (1-4.4)
Eos (Eosinophils)	0.06 x10^9/L (0.1-0.7) Low
Hgb (Hemoglobin)	129 g/L (120-160)
WBC (White Blood Cells)	4.02 x10^9/L (4-11)
RBC (Red Blood Cells)	4.36 x10^12/L (4-5.4)
Hct (Hematocrit)	0.388 L/L (0.36-0.54)
MCV (Mean Corpuscular Volume)	89.1 fL (76-96)
MCH (Mean Corpuscular Hemoglobin)	29.5 pg (27-32)
MCHC (Mean Corpuscular Hemoglobin Concentration)	331 g/L (320-350)
RDW (Red Cell Distribution Width)	10.9 % (11.5-14.5) Low
NRBC (Nucleated Red Blood Cells)	0.00 x10^9/L (0-3)
Platelet	194 x10^9/L (150-400)
NRBC	0.00 % (0-5)
MPV (Mean Platelet Volume)	8.9 fL(6.3-10.3)
Alk Phos (Alkaline phosphatase)	85 U/L (40-150)
Total Protein	70 g/L (64-83)
Potassium	4.1 mmol/L (3.5-5.1)
Sodium	138 mmol/L (136-145)
Chloride	107 mmol/L (98-107)
Glu F (Fasting Glucose)	12.5 mmol/L (4.1-5.6) High
Creatinine	57 µmol/L (50-98)
CO2 (Bicarbonate)	22 mmol/L (22-29)
BUN (Blood Urea Nitrogen)	4.4 mmol/L (3.5-7.2)
ALT (Alanine Aminotransferase)	25 U/L (5-55)
AST (Aspartate Aminotransferase)	16 U/L (5-34)
AGAP (Anion Gap)	13 mmol/L (7-15)

These results indicated normal liver function, normal immunoglobulin levels, and mildly low neutrophil and eosinophil counts, likely reflecting the chronic inflammatory nature of her condition. The patient’s elevated fasting glucose level was attributed to her diabetes.

The patient was diagnosed with VVO-LP by both the Dermatologist and Oral Medicine Consultants and initially treated with dexamethasone mouthwash (1 mg/ml), to be used at least three times daily for two weeks before meals. She was instructed to gargle softly for at least 2-3 minutes, then spit out and refrain from eating or drinking for at least 5-8 minutes to ensure sufficient mucosal absorption. This regimen was to be followed for 7-10 days. After initial satisfactory improvement of the oral ulcers, the lesions recurred two weeks after stopping the mouthwash. She was subsequently referred to a dermatologist on March 16, who prescribed Prednisolone (40 mg) tablets once per day for one week (gradually tapering to a maintenance dose of 5 mg for six months). A skin rash was noticed by the patient after using the medication. Dermovate ointment twice daily and Protopic ointment 0.1% twice daily on weekdays were prescribed. Despite short-term benefits, the patient continued to experience pain, and on March 31, she received intralesional injections of Triamcinolone acetonide 40 mg in 1 ml, diluted with local anesthesia in both buccal mucosa and on the dorsum of the tongue, which provided complete relief from oral ulcers. However, the condition worsened after two weeks. Systemic treatments with various agents were prescribed by the Dermatologist/Immunologist at another hospital. Additionally, she was seen by a gynecologist to treat the vaginal ulcers; no biopsy from the vagina was taken, and diagnosis was based on clinical symptoms of VVO-LP. Treatment modalities included different doses of Cyclosporine and Azathioprine, with symptoms recurring after discontinuation of all used drugs approximately two weeks later. With Isotretinoin 30 mg (last dose in 2020), the patient noticed a 70% improvement in her oral ulcers, skin, and genital symptoms during the four months of use, with a relapse occurring in December 2022. She was referred to a psychologist to evaluate her emotional status, as the ulcers in the mouth, skin, and vagina had impacted her marital relationship and social activities, and was prescribed an antidepressant. Physicians have prescribed different doses of medications; Dapsone was prescribed by her dermatologist, which she couldn’t tolerate as it exacerbated her symptoms and was discontinued immediately due to severe stomach pain and upset. She was also admitted twice to the ER suffering from UTIs and advised to consume more fluids and red fruit juices along with the necessary antibiotics. Lately, the patient stated that she felt an improvement by using Isotretinoin during the medication course and by the Dexamethasone intralesional injections every four weeks.

## Discussion

OLP is a common inflammatory disorder that affects the stratified epithelia of the oral mucosa and occurs with a prevalence of 0.5% to 2.2% in the adult population. It typically appears in the fifth to sixth decades of life and is twice as common in women as in men. It is considered an autoimmune disease in which CD8 T-cells target basal epithelial cells, causing subsequent apoptosis. VVO-LP presents a rare condition that has been reported in the literature only in case report form. In about 20% of cases with OLP, lesions develop in the genital area. Proper diagnostic criteria are still lacking, making disease control and management challenging. Yet, several clinical diagnostic attempts have been published in the literature [5,7].

Simpson et al. proposed an international electronic-Delphi consensus exercise to characterize erosive LP affecting the vulva (ELPV). It consists of a subjective formal feedback process. Consensus was achieved for certain ‘supportive’ diagnostic criteria, including but not limited to erosions/erythematous areas in the vagina, Wickham striae in the surrounding skin, pain/burning, scarring, signs of basal cell degradation, and more. A consensus agreed that the presence of three supportive features makes the diagnosis of ELPV [14].

Other attempts to characterize VVO-LP are still limited, since the rarity of the cases and the diversity of clinical presentations make it difficult to categorize the disease entity. This affects the treatment planning process and broadens intervention modalities which may not benefit affected individuals in the long term.

Clinically, LP presents in three forms: erosive, classic, or hypertrophic. Erosive LP usually affects nonkeratinized squamous epithelium, while classic and hypertrophic LP occurs in keratinized skin and has a diversity of appearances, presenting a clinicopathologic challenge for clinicians. Two cohort studies of vulvar LP that specified clinicopathologic subtypes noted that 6% to 29% of cases were hypertrophic and 4% to 6% were classic [1].

Erosive LP manifests as well-demarcated glazed erythema, often with a hyperkeratotic border. Classic LP is characterized by pruritic papules and plaques of variable color that occur anywhere and spontaneously resolve, while hypertrophic LP is usually characterized as thick violaceous plaques on extensor surfaces of lower extremities and perianal skin [1].

The clinical presentation of OLP may differ between individuals and within the same individual, as various morphologies may occur in the same patient at the same time. Wickham's striae can be found with or without erythema or erosions. Large hyperkeratotic plaques affecting the buccal mucosa and the dorsum of the tongue could also be present [2].

The patient in this case presented with bilateral red and white lesions affecting the buccal mucosa and the dorsum surface of the tongue, characteristic findings of OLP, in addition to ulcerations in the vulvo-vaginal area. The resistance to any systemic medication and the recurrence of ulcers reflect a typical presentation of VVO-LP.

Definitive diagnoses were based on the clinical presentation of mucosal lesions and confirmed by biopsy and histology readings. Additional laboratory testing to rule out systemic disease was also performed in this case.

In a study conducted by Olszewska M et al. to analyze the prevalence of LP lesions in various anatomical locations in patients with VVO-LP, they reported that 63% of included patients had skin involvement, 31% had oral lesions, and 100% of participants had desquamative gingivitis [4].

In this case report, a multidisciplinary approach was assigned for the patient to be seen by Oral Medicine/Dermatologist/Immunologist/Gynecologist and Psychologist as well. A range of medications were used, such as Dexamethasone oral mouthwash which gives initial improvement but with recurrence after therapy was discontinued. Prednisolone 40 mg was prescribed, along with Dermovate ointment and Protopic ointment for skin ulcers. Topical and systemic immunosuppressants used also gave transient improvement but not definitive resolution. The patient continued to be treated with palliative agents to reduce the symptoms.

In a literature review by Ho JK et al., which investigated 50 reports including 424 patients on the treatment of ELP, he found that 27% of the cases were treated with systemic corticosteroids (Prednisone in 92% of them), which resulted in partial or complete resolution of oral lesions. However, treatment outcomes varied among patients with some cases having no benefit, and the average main outcome was only partial improvement [17].

In the same study, Ho JK et al. reported that systemic corticosteroids did not result in complete resolution of vaginal lesions, and multiple recurrences after treatment discontinuation were detected, as well as adverse effects such as oral candidiasis and euphoria, which suggests that corticosteroids are not a long-term therapy for ELP [17].

Systemic retinoids were the second most used treatment in cases of ELP. However, no benefits were reported, especially with acitretin, which yields the poorest outcome, with the best possible outcome being transient resolution and temporary pain relief when using etretinate and isotretinoin. Discontinuation of treatment returns the cases to the pretreatment state or even worse. Immunosuppressant drugs such as azathioprine, thalidomide, and cyclosporine were used most often, with azathioprine appearing to be the most effective, achieving complete resolution with no recurrence up to nine months after therapy discontinuation. However, numerous adverse effects have been reported, which undermine the efficacy of immunosuppressant agents in ELP treatment [17].

Promising results have been reported in the scientific literature when a combination of levamisole and prednisolone was used, leading to the complete resolution of lesions with no recurrence within nine months of follow-up and minimal adverse effects. However, with only one or two publications reporting this, its approval as a definitive treatment remains questionable. Further publications and randomized controlled trials are needed to evaluate the efficacy of such treatments [17].

VVO-LP remains a challenging condition to treat due to the limited and temporary effects of current therapies and high recurrence rates. The incomplete understanding of its etiology and the side effects of certain drugs restrict their long-term use. This case highlights the need for better evaluation of treatment options and a collaborative approach among specialists for improved patient outcomes.

## Conclusions

This case of VVO-LP underscores the complexities in managing this chronic inflammatory condition. Although corticosteroids provided relief, the recurrence of lesions after discontinuation indicates the need for sustained maintenance therapy. Systemic treatments showed only temporary benefits, suggesting that more aggressive or extended treatment strategies may be required. Regular monitoring for potential malignancy is essential, and a multidisciplinary approach involving dermatologists, oral medicine specialists, and gynecologists is key for comprehensive care. Further research into new therapeutic agents and long-term strategies is crucial for improving outcomes in this rare condition. Persistent symptoms were managed with additional interventions like triamcinolone acetonide injections to control pain.
